# Drug-Induced Gingival Overgrowth—Molecular Aspects of Drug Actions

**DOI:** 10.3390/ijms24065448

**Published:** 2023-03-13

**Authors:** Agnieszka Droździk, Marek Droździk

**Affiliations:** 1 Department of Interdisciplinary Dentistry, Pomeranian Medical University in Szczecin, Powstancow Wlkp 72, 70-111 Szczecin, Poland; 2 Department of Pharmacology, Pomeranian Medical University in Szczecin, Powstancow Wlkp 72, 70-111 Szczecin, Poland

**Keywords:** gingival overgrowth, drug-induced gingival overgrowth, gingiva, drug side effects

## Abstract

Drug-induced gingival overgrowth (DIGO) is one of the side effects produced by therapeutic agents, most commonly phenytoin, nifedipine and cyclosporin A. However, the precise mechanism of DIGO is not entirely understood. A literature search of the MEDLINE/PubMed databases was conducted to identify the mechanisms involved in DIGO. The available information suggests that the pathogenesis of DIGO is multifactorial, but common pathogenic sequelae of events emerge, i.e., sodium and calcium channel antagonism or disturbed intracellular handling of calcium, which finally lead to reductions in intracellular folic acid levels. Disturbed cellular functions, mainly in keratinocytes and fibroblasts, result in increased collagen and glycosaminoglycans accumulation in the extracellular matrix. Dysregulation of collagenase activity, as well as integrins and membrane receptors, are key mechanisms of reduced degradation or excessive synthesis of connective tissue components. This manuscript describes the cellular and molecular factors involved in the epithelial–mesenchymal transition and extracellular matrix remodeling triggered by agents producing DIGO.

## 1. Introduction

Gingival overgrowth ascribed to drug-related activity was first associated with the use of phenytoin, an antiepileptic agent introduced into clinical applications in 1937. The first report on gingival overgrowth caused by chronic administration of the drug was published in 1939 [1]. The condition is related to pathological growth of gingival tissues due to excessive expansion of the extracellular matrix, cellular hyperplasia and/or hypertrophy. However, molecular mechanisms involved in the pathology of drug-induced gingival overgrowth (DIGO) have not yet been fully determined. As of today, many other drugs belonging to different therapeutic groups than antiepileptic agents have been confirmed to produce DIGO. Phenytoin, cyclosporine and nifedipine are the drugs most commonly associated with DIGO [2]. The median time-to-onset of DIGO for immunosuppressant, calcium channel blocker and anticonvulsant use is reported to be 71, 262 and 37 days, respectively [3]. The prevalence of DIGO in cross-sectional studies is estimated to be 70% in the case of phenytoin (30% for other anticonvulsant drugs), 30% for nifedipine, 30% for diltiazem, 20% for verapamil and 50–80% for cyclosporine. DIGO usually develops during the first 3 months and reaches a plateau phase at 9 to 12 months [4]. The incidence and prevalence of DIGO according to data from the selected published studies is presented in Table 1.

The list of drugs producing DIGO is not limited to those aforementioned. The wide range of incidence/prevalence suggests many potential mechanisms involved in the pathology, apart from methodological issues (e.g., self-reported, office-diagnosed). In fact, gingival overgrowth is a multifactorial clinical problem with a detrimental role of bacterial plaque interacting with other factors, including drugs. The range of responses can also be ascribed to specific medication regimens, such as cyclosporine. The available information suggests that long-term exposure to a low dose of cyclosporin A (≤200 ng/mL) does not produce effects on the viability or proliferation of human gingival fibroblasts, whereas exposure to high concentrations of the drug (400–800 ng/mL) affect the viability and proliferation [15,16]. However, no correlation between the drug concentrations and DIGO was reported [17,18]. Likewise, the same observations, i.e., dose-dependent responses and no dose-dependent DIGO, were published for phenytoin [19].

**Figure 1:**
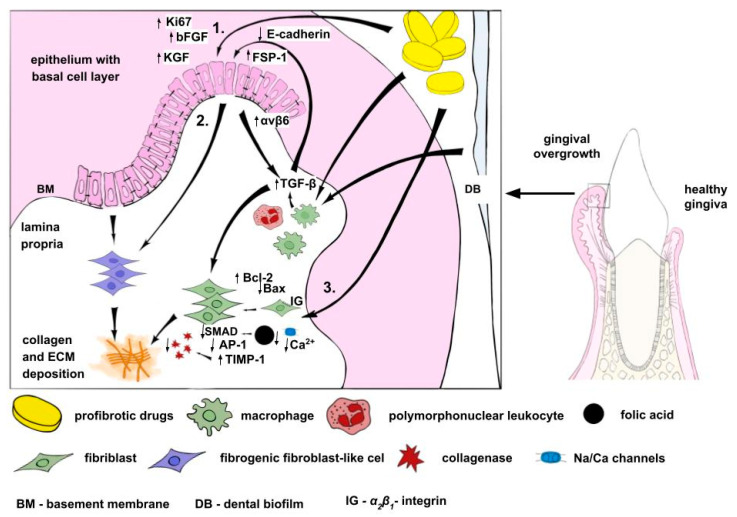
Pathophysiology of drug-induced gingival overgrowth. (1) Profibrotic drugs increase keratinocyte proliferation and decrease keratinocyte apoptosis, accompanied by elevated levels of cell proliferation marker (Ki67), basic fibroblast growth factor (bFGF) or keratinocyte growth factor (KGF) levels. Tumor growth factor (TGF)-β1, a key mediator of epithelial–mesenchymal transition (EMT) in gingival epithelial cells, leads to reductions in epithelial E-cadherin expression and upregulation of fibroblast-specific protein-1 (FSP-1) and αvβ6 integrin levels. Subsequently, gingival epithelial cells lose cell-to-cell contacts. Due to destruction of the basement membrane and lost interactions with the basal gingival surface, epithelial cells undergo multiple biochemical changes that enable them to assume a mesenchymal cell phenotype (fibrogenic fibroblast-like cells), which includes enhanced migratory capacity, elevated resistance to apoptosis and greatly increased production of extracellular matrix (ECM) components. (2) Drugs impact intracellular Ca^2+^ homeostasis (via action on calcium and sodium channels or targeting calcineurin). The decrease in intracellular Ca^2+^ results in reductions in folic acid (FA) uptake and, in turn, reduced intracellular levels of FA, which leads to changes in the activity of matrix metalloproteinases and failure to activate collagenase. TGF-β, apart from the role in EMT, stimulates fibroblastic population activity and increases the relative proportion of sulfated glycosaminoglycans. Decreased cellular FA levels result in reduced expression of SMAD (SMAD), which in turn downregulates the expression of the AP-1 gene. Reduced AP-1 leads to tissue inhibition of metalloproteinase (TIMP)-1 gene expression. An increase in TIMP-1 activity, which is involved in the activation of collagenase, produces a reduction in the amount of activated collagenase. Intracellular collagen processing is reduced via decreased *α*_2_*β_1_*-integrin, which is a specific receptor for collagen type I in fibroblasts, and determines the initial step of collagen phagocytosis.

As stated above, DIGO is most commonly produced by members of sodium channel blockers (phenytoin), calcium channel blockers (nifedipine) and calcineurin inhibitors (cyclosporine). Therefore, those mechanisms, especially inhibition of intracellular cation influx (sodium, calcium), are postulated to play a pivotal role. However, not all drugs affecting intracellular cation status can produce DIGO, and even conversion from cyclosporin A to tacrolimus (calcineurin inhibitors) [20] or from amlodipine to benidipine (calcium channel blockers) [21] resulted in DIGO resolution. In the complex pathophysiology of DIGO, mutual interactions between cellular and extracellular components are modulated by age, genetic predisposition, pharmacokinetic variables, alterations in gingival connective tissue homeostasis, pre-existing dental plaque and gingival inflammation, all of which determine the development of drug-induced lesions [22]. The DIGO pathology is likely to be driven primarily by indirect effects of drugs that interact with innate and acquired immune responses mediated by a vast array of cytokines. Disturbed immune system functions associated with tissue specific responses of gingival cells can drive epithelial–mesenchymal transition (EMT) and extracellular matrix (ECM) remodeling, which result in the observed tissue abnormalities (Figure 1).

## 2. Gingival Morphology in DIGO

The histological picture of DIGO includes cellular changes, characterized by hyperplasia in the junctional epithelium and hypertrophy in keratinized epithelium, which specifically develops in the gingiva. A classic feature of DIGO includes epithelial rete peg extension deep into the connective tissue compared with normal gingival tissues with fibrotic or expanded connective tissues. The lamina propria shows collagen fibrosis with variable fibroblasts, infiltration of inflammatory cells (plasma cells and lymphocytes), increased vascularity, and an amorphous ground substance with evident changes in glycosaminoglycans (GAGs) [23,24]. Patients who suffer from DIGO do not develop fibrosis in other organs, owing to unique signal transduction pathways operating in gingival fibroblasts that confer relative resistance to the effects of some inflammatory mediators, such as prostaglandin E (PGE)2, which could contribute to the tissue specificity [25].

The histopathological evaluation of gingival structures from patients under phenytoin, nifedipine and cyclosporine medications also suggests different, drug-related effects. DIGO produced by phenytoin is characterized mostly by fibrotic changes; cyclosporine activity results in highly inflamed lesions with little fibrosis; and nifedipine induces mixed picture of fibrosis and inflammation [26]. These various responses in the DIGO pathogenic process are suggested to be dependent on genetically predetermined gingival fibroblasts, more avidly responding to drugs inducing gingival overgrowth than other fibroblast subpopulations. The fibroblast heterogeneity, i.e., more or less inducible populations, determines variable behavior in the production of potentially proliferative and fibroblastic cytokines/growth factors and their environmental responses related to ECM components [27].

## 3. Cells in DIGO

### 3.1. Fibroblasts

Specific changes in the gingival connective tissue observed in the course of DIGO development are associated with cellular alterations, finally leading to EMT (Table 2). During EMT, polarized epithelial cells undergo multiple biochemical changes that enable them to assume the mesenchymal cell phenotype (fibrogenic fibroblast-like cells), which includes enhanced migratory capacity, elevated resistance to apoptosis and greatly increased production of ECM components. Gingival epithelial cells, due to destruction of the basement membrane, lose the interaction with their basal surfaces, which results in a disturbed diffusion of factors between connective tissue and the epithelial layers of gingival structures. In general, these pathological features of DIGO are independent of administered drugs, which suggests a common pathway of induction in spite of differences in drugs’ principal modes of action. The deregulated cytokine imbalance seems to play a considerable role in DIGO development, and can support the notion that the direct activity of drugs is not the most important mechanism in DIGO development. 

Cells participating in EMT undergo a transition from having epithelial characteristics to having a mesenchymal cell phenotype, and changes can also affect proliferative capacity and apoptosis (programmed cell death). Exposure of fibroblasts to nifedipine and cyclosporine increases viability and replication [18,28]. These findings correlate with upregulation of the antiapoptotic protein Bcl-2 and downregulation of the proapoptotic protein Bax by cyclosporin A in human gingival fibroblasts that was observed in vitro [29]. These findings suggest also that under nifedipine exposure, elevated levels of miR-4651 targeting the high-mobility group AT-hook 2 (HMGA2) gene inhibits the proliferation of gingival mesenchymal stem cells and arrests the cell cycle at the G0/G1 phase by upregulating cyclin D and cyclin-dependent kinase 2 (CDK2), while downregulating cyclin E through inhibition of HMGA2. Thus, both processes, i.e., decreased cell apoptosis and increased proliferation, can contribute to the development of fibrosis in gingival tissues [30].

The sequel of DIGO events can be triggered by TGF-β1, defined as a key inducer of EMT. Elevated levels of EMT are found in human gingival overgrowth tissues [31,32]. TGF-β is able to induce a variety of effects in epithelial cells, as described below. Epithelial cells gain the ability to migrate in the absence of normal cell-to-cell junctions and damaged basement membranes. The defective basement membrane then permits abnormal communication between the epithelium and the underlying stroma, allowing a shift of biologically active agent levels (e.g., resulting from TGF-β1 stimulation) of fibronectin, fibronectin containing extra domain A (FN-ED-A), connective tissue growth factor (CCN2/CTGF) and lysyl oxidase enzyme activity, which contribute to connective tissue fibrosis [24,31]. The αvβ6 integrin plays a role in the activation of TGF-β1 [32]. It enables cells to interact with the interstitial matrix and to sustain activation of TGF-β1. Fibronectin, and fibroblast-specific proteins (FSP)-1 in particular, are considered to be highly specific markers for fibroblasts [33], impacting the EMT process in DIGO. Elevated FSP-1 levels occur in phenytoin-induced overgrowth in all layers of both the oral and the sulcular gingival epithelia, and indicate vast ECM development through the gingiva [31].

SLUG (also known as SNAIL2) is another possible participant in EMT induced by gingival lesions. It regulates intercellular adhesions between cells [34,35]. In the regulation of Slug, a role of miR-200b was revealed, and administration of cyclosporin A was able to regulate Slug and lower the expression of miR-200b in human gingival fibroblasts [36]. Under exposure of cyclosporin A, a downregulation of miR-200b produced an increase in zinc-finger E-box-binding transcription factor ZEB2 (also known as SIP1-Smad-interacting factor) levels, which is a direct downstream target regulated by miR-200a. Finally, the impact of cyclosporin A on the miR-200/ZEB axis leads to proliferation of normal human gingival fibroblasts [37].

CCN2/CTGF, which elevated expression in both epithelial and connective tissue cells in phenytoin-induced gingival overgrowth tissues, is considered to be a marker of fibroblastic mesenchymal cells [38]. CCN2 promotes the proliferation of various cell types, and is highly expressed in a wide range of fibrotic lesions, such as skin and kidney fibrosis [39,40]. Like other EMT mediators, CCN2/CTGF levels are significantly induced by TGF-β1 in human gingival fibroblasts, and its expression is mediated by JNK and Smad signaling [41,42]. However, CCN2/CTGF expression levels in DIGO might depend on the medication type. The levels of CTGF/CCN2 were found to be the highest in gingival tissues from phenytoin-induced lesions, intermediate in nifedipine-induced lesions, and nearly absent from cyclosporin-A-induced overgrowth structures [26]. These findings highlight possible differences in cellular responses resulting from different drugs producing DIGO.

Fibroblasts activity can be also affected by immune cells (see below) via different mediators. However, some differences between drugs producing DIGO have been reported. This experimental study revealed that cyclosporine alone did not induce evident inflammatory responses, but augmented expression of CD54 (recruit pro-inflammatory cells) and production of IL-6 and IL-8 (induced by TLR2 or TLR4 ligands), whereas phenytoin attenuated those responses in human gingival fibroblasts. Cyclosporine also augmented CD54 expression in the gingiva of mice injected with lipopolysaccharide (TLRs ligand). These results indicate that inflammatory responses of human gingival fibroblasts are modulated positively by cyclosporine and negatively by phenytoin [43]. Interleukin(IL)-1β and IL-6 are proposed to be involved in cyclosporin A-induced gingival overgrowth. Both are highly expressed in the fibroblasts of overgrown gingival connective tissue [44] or gingival crevicular fluid [45] from patients under immunosuppressive therapy. The genetic variant, i.e., polymorphism, of the *IL1A* gene was suggested to be associated with gingival overgrowth in renal transplant patients medicated with cyclosporin A (thus explaining inter-individual susceptibility). Carriage of the *IL1A*(−889)T allele was significantly associated with a reduced risk of DIGO, but not significantly associated with IL-1α protein levels, in gingival crevicular fluid [46]. However, those observations were not replicated in larger populations nor in subjects of different ethnicities, and therefore should be considered as preliminary. No association between *IL6*(−174 G/C) gene polymorphism and gingival overgrowth was observed in renal transplant recipients under cyclosporin A-based immunosuppressive maintenance regimens [47].

Some studies have also explored other potential mediators affecting the function of gingival fibroblasts, but the evidence is scarce (in comparison to TGF-β1), and in most studies, only single agents were reported. Angiotensin II could potentially be another player in DIGO. It can operate in concert with TGF-β. The expression of receptors for angiotensin II, namely, AT1 and AT2, was evidenced in rabbit gingival fibroblasts [48]. In gingival fibroblasts, angiotensin II upregulates TGF-β1 expression through the AT1 receptor [49], which is mandatory for the induction of fibrosis [50]. TGF-β1 can directly stimulate AT1 receptor expression through TGF-β type I receptor kinase (ALK5) and Smads 2/3/4 (possibly in autocrine loop) [51]. Angiotensin II levels in gingival tissue samples are significantly elevated in patients administered nifedipine with developed gingival overgrowth [52], which is in line with experimental findings showing upregulation of angiotensin II production in cultured guinea pig gingival fibroblasts exposed to the drug (as well as phenytoin) [53]. Angiotensin II also modulates renal cell growth and extracellular matrix synthesis by upregulating two significant growth factors, namely TGF-β and CCN2/CTGF, which are also known to contribute to DIGO [54]. The possible involvement of epidermal growth factor (EGF) in DIGO was also suggested. EGF controls cell migration, which is an important phenomenon in tissue formation and remodeling. It promotes wound healing by stimulation of macrophages, fibroblasts migration and synthesis of matrix proteins, and was evidenced in overgrown gingival tissues [55]. Insulin-like growth factors (IGF)-1 and -2 stimulate collagen synthesis and downregulate collagenase activity. In cyclosporine-induced gingival overgrowth tissue, elevated mRNA expression levels of IGF binding protein (IGFBP-5), an IGF-1 response enhancer, were associated with increased collagen and fibrosis [56]. However, some contradictory observations are also available, since a study on rat gingival cells revealed that cyclosporin A stimulated proliferation, but without direct contribution of IGF-1 and IGFBPs (-2, -3, -5, -6). The study suggested, rather, a role of TGF-β1 and FGF-2 in cyclosporine-induced gingival overgrowth [57].

### 3.2. Keratinocytes

A typical feature of DIGO includes disturbed epithelial structures with keratinocyte proliferation, destruction of the lamina propria and rete peg extension deep into the connective tissue in comparison with normal gingival tissues. Keratinocytes demonstrate the same behavior with fibroblasts. Drugs producing DIGO, i.e., phenytoin, nifedipine and cyclosporin A, stimulate the proliferative capacity of keratinocytes, accompanied by elevated levels of a cell proliferation marker, Ki67, and basic fibroblast growth factor (bFGF) or keratinocyte growth factor (KGF) in gingival hyperplasia [58,59,60,61,62]. However, gingival hyperplasia in nifedipine or cyclosporin A-induced gingival overgrowth was not related to enhanced keratinocyte proliferation, but to prolonged cell life [63,64,65,66]. Studies also provide evidence of functional changes in keratinocytes involved in DIGO. Phenytoin-induced human gingival overgrowth tissues revealed diminished epithelial E-cadherin expression, as well as upregulation of fibroblast-specific protein-1 (FSP-1) and αvβ6 integrin levels [31]. E-cadherin expression changes were the most prominent in the basal layers of the epithelium, adjacent to the basement membrane, suggesting that the most advanced transitions to the mesenchymal phenotype in epithelial cells occur at or near the basement membrane. Decreases in E-cadherin expression result in the loss of cell-to-cell contacts and affects epithelial tissue barrier function. Like fibroblasts, keratinocytes respond to elevated levels of TGF-β1. TGF-β1 is a mediator of EMT in epithelial cells, which, upon exposure, show common phenotypic changes, e.g., decreases in E-cadherin and fibronectin; disassembly of cell junctions; and increased levels of matrix metalloproteinases (MMP)-2, -9 and 13, which can contribute to the disturbed barrier function [31,67]. In vitro findings have provided evidence that TGF-β1 exposure leads to reductions in cell surface E-cadherin expression in primary human gingival epithelial cells, which is associated with inhibited barrier function as well as an increase in levels of the aforementioned EMT marker, i.e., CCN2/CTGF (downstream target of TGF-β signaling) [31]. TGF-β1 can also produce other in vitro changes, e.g., an increase in SLUG (zinc-finger transcription factor of the Snail/Slug zinc-finger family). SLUG (also known as SNAIL2) is another possible participant in EMT in gingival lesions. It regulates the disruption of desmosomes at the onset of cytokine-induced EMT and inhibits desmoplakin and desmoglein, in addition to repressing E-cadherin gene expression, and thus promotes the progression of EMT via disruption of tight and desmosomal junctions and intercellular adhesions between cells [34,35]. TGF-β1-induced SLUG expression seems be of importance in the early stages of lesion development and the EMT process in gingival epithelial cells [31]. It is proposed that keratynocyte functions can be also affected by other growth factors. Immunohistochemical staining of gingival tissue/gingival keratinocytes in the oral gingival epithelium of cyclosporin A-induced overgrown gingiva revealed more pronounced expression of EGF receptors as compared to clinically healthy gingival specimens [50].

### 3.3. Immune Cells

There is also an aspect of other cellular actions participating in DIGO development. Cyclosporin A, by forming a heterodimer complex consisting of the drug and its cytoplasmic receptor protein–cyclophilin (which binds calcineurin and inhibits its phosphatase activity), controls immune cell function in the local gingival environment and functional specificities of gingival fibroblasts (fibrosis is a severe complication of cyclosporine therapy in the kidney; the gingiva responds in a different, less fibrotic way). Cyclosporine dampens IL-2-dependent lymphocyte T clonal expansion, i.e., acquired immune response, which in gingival tissues seems to co-operate with innate immune responses (contrary to the kidney, in which the acquired immune response plays a crucial role). In the gingival structures, the innate immune system is particularly important due to microbial and physical insults, and disruption of its functional partner (i.e., acquired immunity) might produce more prominent local cellular responses. Different responses of the innate immunity to cyclosporin A and phenytoin were evidenced in a study on toll-like receptors (TLRs). TLRs are proteins that play a key role in the innate immune system, being expressed on sentinel cells such as macrophages and dendritic cells, and recognize structurally conserved molecules derived from microbes. It was found that cyclosporine alone did not induce evident inflammatory responses, but did augment the expression of CD54 (recruitment of pro-inflammatory cells) and production of IL-6 and IL-8 induced by TLR2 or TLR4 ligands, whereas phenytoin attenuated those responses in human gingival fibroblasts. Cyclosporine also augmented CD54 expression in the gingiva of mice injected with lipopolysaccharide (TLRs ligand). These results indicate that the inflammatory responses of human gingival fibroblasts are modulated positively by cyclosporine and negatively by phenytoin [43]. Macrophages’ contribution to DIGO appears in clinical observations and in vitro studies. Phenytoin-treated individuals exhibit high levels of platelet-derived growth factor (PDGF)B expression, paralleled by a significant increase in the inflammatory macrophage phenotype in hyperplastic gingival tissue, and a significant increase in the reparative/proliferative macrophage phenotype in severely inflamed tissues [68]. Cyclosporin A is also able to induce PDGF expression in human gingival fibroblasts, and thus to control EMT and cellular responses. Macrophages originating from EMT were identified as PDGF B-producing cells, and were demonstrated to be distributed in a non-uniform manner throughout the papillary connective tissue of patients receiving cyclosporin A therapy and exhibiting gingival overgrowth [69]. Different levels of PDGF can also be used to explain the histopathological picture of DIGO, i.e., more potent induction of profibrotic factors by phenytoin (TGF-β1, PDGF) than by nifedipine and cyclosporine (PDGF) [70].

## 4. Extracellular Matrix in DIGO

### 4.1. Collagen

The fibrotic appearance of the gingival tissues in DIGO is determined by an imbalance between the synthesis and degradation processes leading to excessive accumulation of ECM proteins, including collagen type I. The mature collagen in its covalent bonds is insoluble in ECM, and is deposited. Molecular and biochemical mechanisms that trigger this pathological process have not been completely elucidated, and contradictory reports are available. However, the observations can be applied to construct possible pathophysiological events in line with clinical findings (Table 3). The imbalance that leads to DIGO might be related to decreased collagen degradation, not to an increase in its synthesis. The following findings support this hypothesis, i.e., decreased collagen production in vitro [71], reduction in mRNA expression of collagen type I and III coding genes associated with higher density of these fibers [72], inhibition of ECM production by gingival fibroblasts and cell proliferation in vitro, reduced hydroxylation of proline residues on collagen by inhibition of prolyl-3-hydroxylase activity and formation of collagen triple-helical structure in the endoplasmic reticulum during its biosynthesis and, thus, reduced fibrosis [73]. Therefore, impaired ECM degradation may play a pivotal role in DIGO. In these processes, direct effects of the most common DIGO drugs, i.e., antiepileptics, immunosuppressants and calcium channel blockers, are proposed to be involved. All of these agents impact intracellular Ca^2+^ homeostasis. Calcium channel blockers antagonize the activity of membrane L-type calcium channels; cyclosporin A interacts with intracellular levels of calcium by targeting calcineurin; and phenytoin blocks sodium channels, which affects the function of the membrane sodium–calcium exchanger. Additionally, phenytoin can increase intracellular calcium levels by acting on calcium-permeable ion channels (TRPA1), transient receptor potential channels of the vanilloid subtype (TRPV1), and its capsaicin-insensitive isoform TRPV1b. Those channels were determined to be expressed in human gingival fibroblasts [74]. The decrease in intracellular Ca^2+^ results in a reduction in folic acid uptake [75]. This reduced levels of folic acid leads, in turn, to changes in the metabolism of MMPs and the failure to activate collagenase [76]. TGF-β seems to be a crucial regulator of ECM formation. Increased TGF-β levels in gingival tissues induced by many drugs producing DIGO, apart from their role in EMT, can be associated with stimulation of fibroblastic population activity. It promotes activity of lysyl oxidase, a copper-dependent extracellular enzyme produced by fibrogenic cells, which catalyzes the final enzymatic step of post-translational modifications required for the cross-linking of collagen and elastin in the synthesis of the functional extracellular matrix by human gingival fibroblasts, i.e., conversion of soluble monomers of collagen and elastin into insoluble fibers in the extracellular matrix [48]. The enzyme expression was reported to be increased in cyclosporin A-induced gingival overgrowth specimens, and it increased with the grade of inflammation [77]. TGF-β can also induce gene expression and protein synthesis of CTGF/CCN2 in human gingival fibroblasts, but TGF-β and CTGF/CCN2 appear to promote collagen accumulation by mostly independent mechanisms in human gingival cells [48,78]. CTGF/CCN2 have a variety of biological activities (see above for a description of its cellular effects). CTGF expression was significantly higher in phenytoin-induced gingival overgrowth than in cyclosporin A- or nifedipine-induced gingival overgrowth, and accordingly higher in the presence of more than fewer fibrous lesions [26,79]. CCN2 expression correlates with fibrotic conditions and appears to contribute to collagen deposition in these processes, e.g., mice bearing a fibroblast/smooth muscle cell-specific deletion of CCN2 expression in dermal fibroblasts are resistant to bleomycin-induced skin fibrosis [80]. It was determined that binding of CTGF with integrin α_6_β_1_ results in the accumulation of insoluble collagen in gingival human fibroblast cultures and eventually stimulates ECM production [81]. TGF-β over-expression was also related to an increase in the relative proportion of sulfated glycosaminoglycans (GAGs) (heparan sulfate, dermatan sulfate and chondroitin sulfate) retained in the cellular fraction in cultured human gingival fibroblasts treated with cyclosporin A. These changes were associated with alterations in the fine structure of GAGs, which showed a decrease in alpha-l-iduronic acid content [82].

A shifted balance in the extracellular matrix towards the promotion of fibrosis can also be determined by reduced degradation processes. Components of the matrix, mainly collagen fibers, can be degraded by the extracellular pathway related to secretion and activity of collagenases, as well as intracellular processing of collagen via phagocytosis by fibroblasts. The accumulation of collagen seen in gingival overgrowth can be explained by the inhibition of collagenolytic activity within the gingival tissues, which is induced by cyclosporine [83]. Collagenase synthesis, activation and functional form and release is a multi-step process which seems to be determined by calcium influx regulating, among others, folic acid uptake. As stated above, drugs producing DIGO reduce calcium influx, then folic acid uptake, and, finally, the synthesis and activation of collagenases [76]. Decreased cellular folic acid levels result in reduced expression of SMAD (signal transducer proteins and transcriptional modulators that mediate multiple signaling pathways), which in turn downregulate expression of the AP-1 gene. Reduced AP-1 leads to the activation of tissue inhibitors of metalloproteinase *TIMP1* gene expression [84,85]. An increase in TIMP-1 activity, which is involved in the activation of collagenase, produces a reduction in the amount of activated collagenase. TIMP-1 also controls the activity of other proteases. There was a reduction in the gene expression of MMP-1, -2 and -3 by phenytoin administration, while increase in the TIMP-1 mRNA in human gingival fibroblasts was reported [72]. Similar activity was also evidenced for cyclosporin A, i.e., inhibition of MMP-1 expression at both the mRNA and protein levels in a dose- and time-dependent manner, but inhibition of *TIMP1* gene expression (with no effects on TIMP-1 protein levels) [84]. This activity of cyclosporin A can also be attributed to the regulation of SMADs. The drug produced Smad3 activation and suppression of miR-29b, which is a downstream inhibitor of TGF-β/Smad3-mediated fibrosis in rat gingival fibroblasts [86].

In DIGO, not only is extracellular degradation of collagen reduced, but its intracellular processing is as well. *α_2_*β*_1_*-integrin functions as a specific receptor for collagen type I in fibroblasts, and determines the initial step of collagen phagocytosis providing adhesive interaction between fibroblasts and collagen [87]. It was reported that phenytoin significantly decreased collagen endocytosis, which was associated with lower expression of *α_2_*β*_1_*-integrin [71]. An experimental study in rats exposed to cyclosporine demonstrated decreased integrin expression in fibroblasts derived from gingival overgrowth tissues [88]. The genetic polymorphism in α2-integrin gene 807T/C can contribute to a predisposition to DIGO, since the α2 +807C allele was documented to be a genetic risk factor for gingival overgrowth [89]. Cyclosporin A also reduces levels of β1- integrin as well as discoidin domain receptor 1 (DDR1) (fibrillar collagen is its primary ligand) in human fibroblasts obtained from gingival explants. These effects of cyclosporin A may lead to reduced collagen degradation by fibroblasts, and in turn to a net increase in tissue collagen and gingival overgrowth [90].

Deficits in the activity of cathepsins can also be associated with DIGO. Cathepsins are lysosomal cystein proteinases responsible for the digestion of up to 90% of long-lived cellular proteins [91]. Therefore, impaired cathepsin activity may lead to excess accumulation of extracellular matrices in fibrotic diseases. Mice deficient in the cathepsin-L gene were found to have enlarged gingiva, similar to gingival overgrowth. Histological observation of the gingiva demonstrated typical features of acanthosis, a phenotype very similar to that of experimentally induced gingival overgrowth [92]. Nifedipine, cyclosporine and phenytoin selectively inhibited cathepsin-L activity (but not that of cathepsin-B) and mRNA expression in cultured gingival fibroblasts [92,93]. In addition, it was evidenced that the cAMP-response element binding protein (CREB), the phosphorylation of which is regulated by calcineurin (phosphatase, cyclosporine target), is engaged in cyclosporin A-mediated downregulation of cathepsin L synthesis in human gingival fibroblasts [94,95].

### 4.2. Glycosamnoglycans (GAGs)

Fibroblasts under activity of TGF-β, bFGF, PDGF and CTGF, as well as other biological molecules, modify the aspects of the biochemical composition of ECM, e.g., activity of lysyl oxidase or prolyl-3-hydroxylase (also see above), thus increasing the total quantity of GAGs, periostin and fibronectin [26,73,96]. Elevated extracellular levels of low-molecular-weight hyaluronan, which is a linear non-sulfated GAG providing structural and functional support to cells, are found in human gingival fibroblast cultures exposed to cyclosporin A as a consequence of the increased expression of hyaluronan synthase 3 and hyaluronidases -1 and -2 [82]. The GAGs under cyclosporin A exposure also showed a decrease in alpha-l-iduronic (IdoA) acid content. The presence of IdoA residues determined interactions with a broad range of proteins, including TGF-β, which may modulate the biological activity [97]. Cyclosporin A was also found to affect levels of sulfated GAGs, which are polydisperse linear polysaccharides composed of alternate units of hexosamine and uronic acid, connected by glycosidic linkages and sulfation at different positions of the sugar moieties. They include proteoglycans such as heparin, heparan sulfate, dermatan sulfate and chondroitin sulfate. Exposure to cyclosporin A resulted in a significant decrease in dermatan sulfate levels in comparison to non-treated normal gingival fibroblasts. The production of heparan sulfate and chondroitin sulfate was not affected by the drug [83]. These changes may affect interactions of sulfated GAGs with a variety of proteins participating in cell–matrix interactions and the activation of cytokines, enzymes and growth factors. Interactions with TGF-β, FGF and vascular endothelial growth factor [98,99] may be of importance in the pathogenesis of DIGO. Elevated levels of immunostaining for heparan sulphate were reported in nifedipine-induced and phenytoin-induced hyperplastic gingival tissues [30]. Studies with phenytoin suggest possible differences in the distribution of sulfated GAGs, with intracellular sulfated GAGs in the attached gingiva and extracellular sulfated GAGs in the free gingiva [100].

### 4.3. Fibronectin

DIGO was also evidenced to be associated with accumulation of fibronectin, which is a non-fibrous protein in ECM. Nifedipine- and phenytoin-induced human gingival overgrowth contains increased levels of fibronectin and alternatively spliced fibronectin extra type III domain A in connective tissue stroma. TGF-β1 was demonstrated to induce the synthesis (and mRNA expression) of fibronectin [36]. Its distribution in ECM could be determined by the type of medication. In phenytoin-induced gingival overgrowth, tissue fibronectin presented as thin fibers with variable lengths and penetrated the basement membrane of the epithelium in immunostaining. Cyclosporin A produced fibronectin of greater lengths and parallel distribution, whereas in the nifedipine-induced gingival overgrowth, a delicate microfibrillar network of fibronectin resulted in a “cloud” pattern of distribution [101]. Fibronectin is a ligand for integrins, of which different distributions in gingival overgrowth lesions may affect protein distribution patterns [102]. The altered status of fibronectin in ECM can also affect cell functions, e.g., its interactions with actin filaments on the fibroblast surface or regulation of gene expression, cell proliferation and cell migration.

### 4.4. Periostin

Periostin, a matricellular protein, is another fibrosis marker identified in DIGO. It was found to be upregulated in gingival connective tissue in nifedipine-induced gingival overgrowth [103], possibly due to induction by TGF-β, elevated levels of which were observed in DIGO by nifedipine. Likewise, phenytoin stimulates the expression of periostin in human gingival fibroblasts. This effect was mediated by phosphorylation of Smad3 [104] resulting from reduced intracellular folic acid levels. Periostin was reported to promote extracellular matrix biosynthesis through its interactions with procollagen-C-proteinase BMP1 proteins, which process many important extracellular matrix protein precursors to mature functional extracellular matrix structures [104].

### 4.5. Other Factors

Some preliminary studies have also suggested the involvement of other factors in the pathogenesis of DIGO. An increased expression of SPOCK1 (osteonectin) in calcium channel blocker-induced gingival overgrowth tissues was reported by Alshargabi et al. [105]. SPOCK1 is an extracellular proteoglycan that induces EMT in several cancer cell lines and exhibits protease-inhibitory activity. Transgenic mice overexpressing Spock1 developed obvious gingival overgrowth and fibrosis phenotypes, and positively correlated with EMT-like changes. SPOCK1 was also shown to interact with TGF-β1 and MMP-9 in in vitro experiments, suggesting its contribution to DIGO.

Gene expression studies (a limitation of which is that they did not provide information about the cycle threshold, nor, thus, mRNA expression levels) suggest that diphenylhydantoin and gabapentin induce a significant upregulation of genes involved in extracellular matrix deposition, i.e., *COL4A1* (collagen and ECM structural constituent), *LAMB3* (basement membrane constituent) and cyclosporin A-*COL7A1* (collagens and ECM structural constituent), in primary fibroblasts [106,107].

## 5. Conclusions

Phenytoin, nifedipine and cyclosporin A are the drugs which most commonly induce DIGO. The pathophysiology of DIGO is complex due to a plethora of interacting factors postulated to be involved. The best-defined steps in DIGO’s sequelae of events include sodium and calcium channel antagonism and intracellular handling of calcium, which in turn lead to intracellular deficiency of folic acid, and then to disturbed cellular functions that promote gingival overgrowth. The epithelial–mesenchymal transition of epithelial cells results in cell proliferation and invasion through the basement membrane and migration into connective tissue stroma, where new extracellular matrix production occurs. However, the details of the processes involved in DIGO still remain to be defined in further studies.

## Figures and Tables

**Table 1:** Drug-induced gingival overgrowth.

Drug	Prevalence/Incidence (%)	Ref.
Antiepileptic drugs		
Phenytoin	65.4	[3]
	57.0	[1]
	20.0	[5]
	50.0	[6]
Lamotrigine	61.0	[6]
Carbamazepine	15.1	[3]
	32.0	[6]
Oxcarbazepine	71.0	[6]
Levetiracetam	6.5	[3]
Phenobarbital	19.7	[3]
	53.0	[6]
Primidone	56.5	[3]
Topiramate	2.9	[3]
Valproic acid	17.5	[3]
	44.0	[6]
Immunosuppressant drugs		
Cyclorporine	39.4	[3]
	22.4	[7]
	44.9	[8]
	30.0	[9]
Everolimus	4.2	[3]
Sirolimus	6.6	[3]
Mycophenolate mofetil	13.1	
Calcium channel inhibitors		
Nifedipine	57.9	[3]
	6.3	[7]
	50.8	[10]
	83.0	[11]
Amlodipine	94.8	[3]
	3.0	[12]
	1.7	[7]
Diltiazem	20.0	[11]
	4.0	[13]
	2.2	[7]
Verapamil	19.0	[14]
Miscellanuous		
Clobazam	65.4	[3]

**Table 2:** Principal cells in DIGO.

Cell Type	Function in Gingival Physiology	Function in DIGO
Epithelial cells/Keratinocytes	keratinocytes, dominant cells of the epidermis (90% of gingival cell population); self-renewal capacity; contribute to gingival defense (desquamation of superficial epithelial cells prevents bacterial colonization)	increased proliferative activity and reduced apoptosis; loss of interactions with lamnia propria and fibroblasts
Fibroblasts	production of collagen and extracellular matrix components in tissue regeneration	conversion to mesenchymal cell phenotype (i.e., fibrogenic fibroblast-like cells, myofibroblasts), production of extracellular matrix components; production of proliferative cytokines and growth factors; reduced collagen degradation activity
Macrophages	innate immunity	production of proliferative cytokines and regulation of extracellular matrix production by fibroblasts
Mast cells	innate immunity	production of proliferative cytokines; vasodilation (due to histamine release)
Polymorphonuclear lymphocytes	innate immunity	interaction with bacteria in dental plaque; proliferative cytokine production

**Table 3:** Extracellular matrix (ECM) in DIGO.

Structure	Function in Gingival Physiology	Function in DIGO
Collagen type I	major structural component of connective tissue; insoluble; preservation of structural integrity and tissue function	decreased degradation and/or increased synthesis; reduced hydroxylated proline content; reduced cross-linking; changes in three-dimensional organization
Elastin	minor structural component of connective tissue (5% of gingival tissue); insoluble	decreased degradation; reduced cross-linking; changes in three-dimensional organization
Fibronectin	non-fibrous protein in ECM; binds to integrins and other ECM proteins such as collagen, fibrin and heparan sulfate proteoglycans; controls cell adhesion, growth, migration and differentiation	increased levels; disturbed interaction of fibroblast actin filaments with integrins; regulation of cell proliferation and cell migration
Periostin	matricellular protein; ligand for integrins; regulates adhesion and migration of epithelial cells	increased levels; promotion of ECM synthesis
Collagenase 1 (MMP-1)	cleavage of peptide bonds in collagen	reduced activity; collagen accumulation
Prolyl-3-hydroxylase	catalysis of prolyl 3-hydroxylation in many collagen types, leading to formation of hydroxyproline	reduced activity; formation of collagen triple-helical structure
Lysyl oxidase	catalysis conversion of lysine into highly reactive aldehydes that form cross-links in ECM proteins	increased activity; reduced collagen and elastin cross-linking
Tissue inhibitor of metallo- proteinase-1	protease inhibitor of matrix metalloproteinases (collagenase 1–MMP-1)	increased activity; reduction in collagenase activation; reduction in metalloproteinases activity
*α_2_β_1_*-integrin	cell receptor for extracellular matrix proteins (collagen)	reduced levels; decreased collagen phagocytosis
Cathepsins	lysosomal cystein proteinases	reduced activity; decreased intracellular collagen degradation
Glycosamnoglycans	structural and functional support for cells	elevated levels; modulation of enzymes, growth factors and cytokine activities

## Data Availability

Not applicable.
